# Baetis (Baetis) cypronyx sp. n., a new species of the *Baetis alpinus* species-group (Insecta, Ephemeroptera, Baetidae) from Cyprus, with annotated checklist of Baetidae in the Mediterranean islands

**DOI:** 10.3897/zookeys.644.10413

**Published:** 2017-01-10

**Authors:** Roman J. Godunko, Tomáš Soldán, Arnold H. Staniczek

**Affiliations:** 1 State Museum of Natural History, National Academy of Sciences of Ukraine, Teatralna 18, 79008 Lviv, Ukraine; 2 Institute of Entomology, Biology Centre, Czech Academy of Sciences, Branišovská 31, CZ–37005 České Budějovice, Czech Republic; 3 Stuttgart State Museum of Natural History, Department of Entomology, Rosenstein 1, 70191 Stuttgart, Germany

**Keywords:** Baetinae, Baetis alpinus species-group, checklist, distribution, endemism, Mediterranean islands

## Abstract

A detailed description of the larvae of Baetis (Baetis) cypronyx **sp. n.**, a representative of the *Baetis alpinus* species-group within the mayfly family Baetidae, is provided, including a differential diagnosis with regard to closely related species of the group, especially *Baetis melanonyx* (Pictet, 1843) and *Baetis baroukianus* Thomas & Dia, 1984. The new species is mainly distinguished by mouthparts (i.e. the shape and setation of labrum, maxillary and labial palps, details of paraglossae and mandibular incisors), setation of legs and abdominal terga, and length of paracercus. All available data on the biology of this putative endemic species of Cyprus are summarized. Annotated distributional data of the 33 species of Baetidae so far recorded from the Mediterranean islands are given, including new records and also including first data from Malta.

## Introduction

The first contribution to the Baetidae of Cyprus (Soldán and Godunko 2008) included the description of two new species from Cyprus and neighbouring island of Rhodos in Greece. *Baetis mirkae* Soldán & Godunko, 2008 of the *Baetis lutheri* species-group was found on both islands, and later considered as East Mediterranean (Pontomediterranean) species (Bauernfeind and Soldán 2012: 124). *Baetis irenkae* Soldán & Godunko, 2008 of the *Baetis buceratus* species-group is so far only known from three Cypriote localities and probably is endemic to Cyprus (Soldán and Godunko 2008: 95, Bauernfeind and Soldán 2012: 167). Two of these localities are in Limassol District (Kryos River at Kalidonia waterfalls and Diplos River at Chantara waterfalls) and were sampled during an extensive survey of aquatic invertebrates in May–June of 2004 (Soldán and Godunko 2008). Both localities revealed a relatively high diversity of benthic insects, namely high abundances of the mayfly genera Epeorus (Ironopsis), *Electrogena*, and *Baetis* (*Baetis* s. str., *Nigrobaetis* Novikova & Kluge, 1987). One species belonging to the *Baetis alpinus* species-group is described below as Baetis (Baetis) cypronyx sp. n.

The *Baetis alpinus* species-group was established by Müller-Liebenau (1969: 46) [i.e. *alpinus*-Gruppe] for three species, namely Baetis (Baetis) alpinus (Pictet, 1843) Baetis (Baetis) melanonyx (Pictet, 1843) and Baetis (Baetis) nubecularis Eaton, 1898. This species-group with Holarctic distribution includes 12 Western Palaearctic species from Europe, Mediterranean, Minor Asia, and North Africa. According to Müller-Liebenau (1969), Jacob (2003), Soldán and Godunko (2009), and Bauernfeind and Soldán (2012), the distinguishing characters for this species-group can be summarized as follows:

***Larvae***: (**i**) body flattened ventrally, with shortened abdomen; (**ii**) segments of antennal flagellum each shortened in the distal two thirds of the antenna; (**iii**) labrum usually with more than 6–7 (up to 22) long, submarginal setae; (**iv**) outer mandibular incisor group roughly triangular and often fused; (**v**) segment 2 of maxillary palp with one or more (sometimes numerous) stout setae on conical protuberance; (**vi**) pronotum with conspicuous dark pattern; (**vii**) sternal protuberances on meso- and metathorax more or less developed, pointed or rounded apically; (**viii**) outer margin of femora with medium or long bristles, acutely pointed or obtuse apically, arranged in 1–3 rows centrally and proximally; (**ix**) tarsal claws with a pair of fine subapical setae; (**x**) abdominal terga generally light, with marked dark spots centrally; (**xi**) posterior margins of abdominal terga with a row of triangular, more or less pointed spines; (**xii**) surface of abdominal terga usually without distinct corrugations, and usually covered with numerous, tongue-shaped, triangular or spatulate scales and their sockets; (**xiii**) paracercus more or less reduced (occasionally strongly reduced).

***Imagines***: (**xiv**) hind wings with three longitudinal veins, cross veins present or absent; (**xv**) abdominal terga relatively dark and translucent; (**xvi**) basal segment of forceps roughly cylindrical or subcylindrical, with inner, more or less expanded, conspicuous apicomedial projection, often forming a distinct rim; (**xvii**) forceps segment 2 subcylindrical, more or less constricted near base; (**xviii**) forceps segment 3 variable, egg-shaped or subcylindrical, nearly 2–3 times longer than wide.

Apart from the description of the new species, additional objectives of this contribution are to discuss its differential diagnosis and its difference to other representatives of the *Baetis alpinus* species-group, to summarise available data on the biology and distribution of the new species, and to present an annotated checklist of the Baetidae in the Mediterranean islands.

## Material and methods

### Material

Most specimens of the new species were collected in the Kryos River at Kalidonian Waterfalls; additional material was collected in Diplos River at Chantara Waterfalls (for numbers of specimens, their proper localities, and deposition see below). Holotype and 45 paratypes of the new species are housed in the Institute of Entomology, BC CAS (České Budějovice, Czech Republic), 22 paratypes in the collection of State Museum of Natural History NASU (Lviv, Ukraine), and 22 paratypes are stored in the Staatliches Museum für Naturkunde (Stuttgart, Germany). Additional paratypes are deposited in the collection of CNR-IRSA Water Research Institute (Brugherio, Italy).

### Morphological study

The specimens were preserved in 70–80% ethanol. Eight paratypes were mounted on slides with Euparal liquid. Drawings were made using a Zeiss Axioplan microscope with a camera lucida. Photographs of larvae were taken using a Leica Z16 APO macroscope and processed with Leica Application Suite™ Version 3.1.8 to obtain combined photographs with enlarged depth of field. Photographs were subsequently enhanced with Adobe Photoshop™ CS3.

Specimens used for SEM were dissected and dehydrated through a stepwise immersion in ethanol and then dried by critical point drying (Leica EM CPD300). The mounted material was coated with a 5 nm Au/Pd layer (Leica EM ACE200) and subsequently examined and photographed with a Zeiss EVO LS 15 scanning electron microscope. SEMs were subsequently enhanced with Adobe Photoshop™ CS3.

### Terminology

Terminology and corresponding acronyms recently proposed for the representatives of the subgenus Rhodobaetis Jacob, 2003 by Godunko et al. (2015) are used to describe body setation (e.g. to characterise types of stout setae and scales). Further acronyms e.g. *FT* (for designation of flat-tipped sensillum), *B* (for sensillum basiconicum) and *Hr* (for hair-like setae) used here have been proposed earlier by Gaino and Rebora (1996, 2003). Additionally, a new type of tongue-shaped scales (*SC-tg*; 7.5−11.0 µm in length) is described and depicted. Morphological characters to distinguish Baetis (Baetis) cypronyx sp. n. from other representatives of *Baetis alpinus* species-group, and especially from closely related *Baetis melanonyx* are given according to Müller-Liebenau (1969), Thomas et al. (1983), Thomas and Dia (1984), Peru and Thomas (2001), Jacob (2003), Kluge and Novikova (2011), Bauernfeind and Soldán (2012) and Sroka et al. (2012). All discriminating characters are summarized in Table 1.

**Table 1.** Morphological characters in Baetis (Baetis) cypronyx sp. n. (Figs 1–4, 5A, 5C, 6–24) *Baetis baroukianus* Thomas & Dia, 1984 (Figs 29–32), and *Baetis melanonyx* (Pictet, 1843) (Figs 5B, 5D, 25–28). Important differences in characters are marked in grey. Quotient *q* was proposed by Sroka et al. (2012), representing the degree of asymmetry of labial palps. * – based on published data and our own larval material.

No.	Character	*Baetis cypronyx* sp. n.	*Baetis baroukianus* Thomas & Dia, 1984*	*Baetis melanonyx* (Pictet, 1843)*
	*Head*			
1.	Setation of clypeus	solitary *FT*, *B*, and *Hr* setae along with their bases	solitary *B* and *Hr* setae along with their base, *FT* setae more abundant	solitary *B* and *Hr* setae along with their base, *FT* setae more abundant
2.	Setation of frons	solitary *FT*, *B*, and *Hr* setae along with their bases	solitary *FT* and *Hr* setae, along with their bases	solitary *FT* and *Hr* setae along with their bases
3.	Setation of scape and pedicel	solitary *FT* and *Hr* setae, only *B* setae more abundant	solitary *FT* and *Hr* setae, only *B* setae more abundant	solitary *FT* and *Hr* setae, only *B* setae more abundant
	*Mouthparts*			
4.	Labrum: shape	distinctly oblong-shaped, nearly rectangular	distinctly oblong-shaped, nearly rectangular	rather oblong-shaped, narrowed proximally
5.	Labrum: mean width/length ratio	1.80–1.88	1.80–1.95	1.75−2.00
6.	Labrum: number of long submarginal setae	1 + 11–18	1 + 19–21 (15–18)	1 + 14–22 (14–21)
7.	Labrum: number of long marginal setae	6–9	6–8	8–12
8.	Mandibles: number of teeth of inner incisor group	3–4	2	1–2
9.	Mandibles: number of teeth on prostheca	8–10	8–10	9–10
10.	Maxillary palps: number of stout setae at the tip of distal segment	1 (occasionally 2)	1	1
11.	Paraglossae: number of regular rows of apical bristles	2	4–5	3
12.	Paraglossae: number of bristles on outer margin	5–10	6–12	8–12
13.	Paraglossae: number of setae on ventral surface	3−5	3–6	4−6
14.	Labial palps: shape of segment 3	nearly symmetrical and evenly rounded	distinctly asymmetrical and conical	nearly symmetrical and evenly rounded
15.	Labial palps: mean width/length ratio of segment 3	1.03–1.07	1.07–1.09	1.30–1.35
16.	Labial palps: number of stout setae on dorsal surface of segment 3	18–25	14–16	22−28
17.	Labial palps: degree of asymmetry [quotient *q*]	0.76–0.88	0.52–0.56	0.82−0.94
	*Thorax and legs*			
18.	Shape of sternal protuberances on meso- and metathorax	prominent, pointed	prominent, rounded	small, rounded
19.	Foreleg tibia/femur length ratio	1.0	0.9−1.0	0.9−1.0
20.	Hind leg tibia/femur ratio	0.9−1.0	0.9−1.0	0.8−1.0
21.	Outer margin of femora: shape of long bristles	bluntly pointed and/or obtuse apically	acutely pointed apically	acutely pointed apically
22.	Outer margin of femora: number of rows of long bristles proximally and centrally	2–3	1	1 (occasionally 2)
23.	Outer margin of femora: shape of submarginal stout setae	*STSm-bp*	*STSm-bp*	*STSe-bp*
24.	Outer margin of tibia: shape of stout setae	*STSm-p*, *STSm-bp*	*STSm-p*, *STSm-bp*	*STSe-bp*
25.	Tarsal claw: number of strong teeth	10–11	12–14	8−11
26.	Tarsal claw: number of rows of marginal teeth	1	1	1
27.	Tarsal claw: two subapical hair-like setae	present	present	present
	*Abdomen*			
28.	Surface of terga: scales	present, not numerous	present, not numerous	present, not numerous
29.	Surface of terga: scales sockets	present, not numerous, often absent on tergum X	present, not numerous, always present on tergum X	present, numerous, always present on tergum X
30.	Surface of terga: shape of scales	*SC-it*, *SC-tg*	*SC-it*, *SC-tg*	*SC-it*, *SC-tg*
31.	Posterior margin of terga II–VIII: shape of spines	triangular, not shortened, some bluntly pointed and some acutely pointed	triangular, shortened, some bluntly pointed and some acutely pointed	triangular, shortened, some bluntly pointed and some acutely pointed
32.	Posterior margin of terga III–VIII (IX): submarginal row of smaller spines	present	absent	absent
33.	Shape of gills I and VII	nearly symmetrical	nearly symmetrical	slightly asymmetrical
34.	Shape of gills II−V	asymmetrical	asymmetrical	asymmetrical
35.	Paraproct plate (inner margin): number of marginal spines	8–12	0–4	7−11
36.	Paraproct plate (inner margin): number of submarginal stout setae	2–8	5–8	8–12
37.	Paraproct plate (inner margin): shape of submarginal stout setae	*STSs-bp*, *STSm-bp*	*STSs-ov, STSm-ov*, occasional *STSs-bp* and *STSm-bp*	*STSs-ov, STSm-ov*, occasional *STSs-bp* and *STSm-bp*
38.	Paraproct plate (surface centrally): type of setation	tiny setae only	tiny setae only	tiny setae only
39.	Paracercus	reduced; 2–16 segments	well-developed (1/2−2/3 of cerci length) or shortened (more than 15 segments)	well developed; 1/2−2/3 of cerci length
40.	Cerci and paracercus: posterior margin of segments	row of broad triangular spines, additional uneven submarginal row of smaller spines	row of broad triangular spines	row of broad triangular spines

**Figures 1–2.**
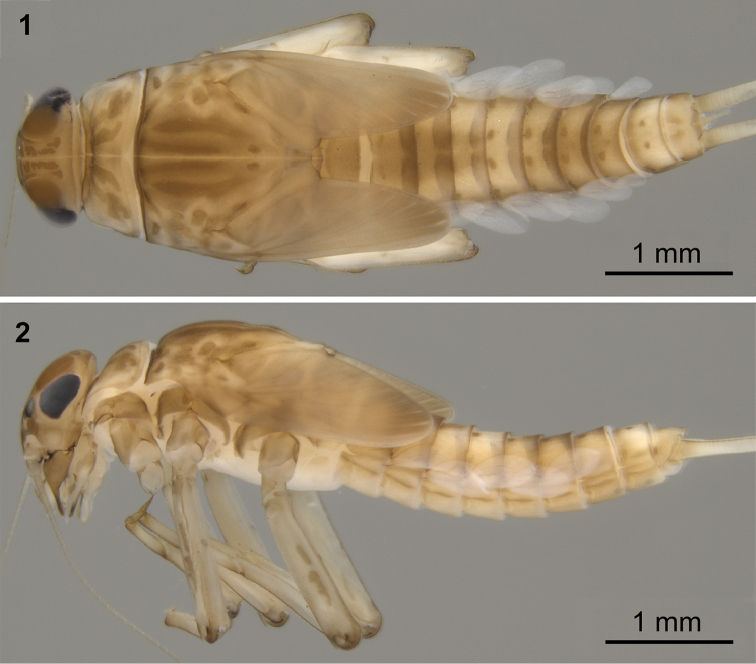
Colour pattern of Baetis (Baetis) cypronyx sp. n., larva, male, paratype (material from type locality): **1** body, dorsal view **2** body, lateral view.

**Figures 3–4.**
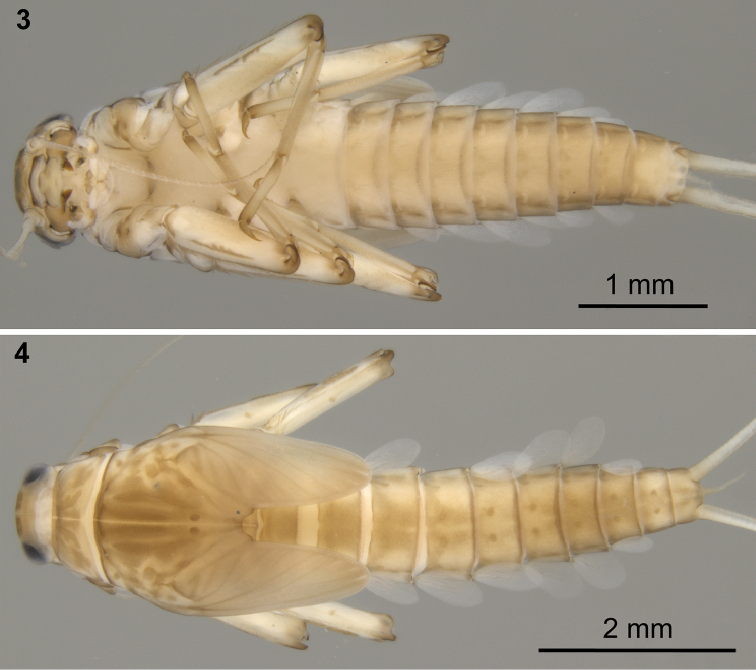
Colour pattern of Baetis (Baetis) cypronyx sp. n., larvae, male (**3**) and female (**4**), paratypes (material from type locality): **3** body, ventral view **4** body, dorsal view.

**Figure 5.**
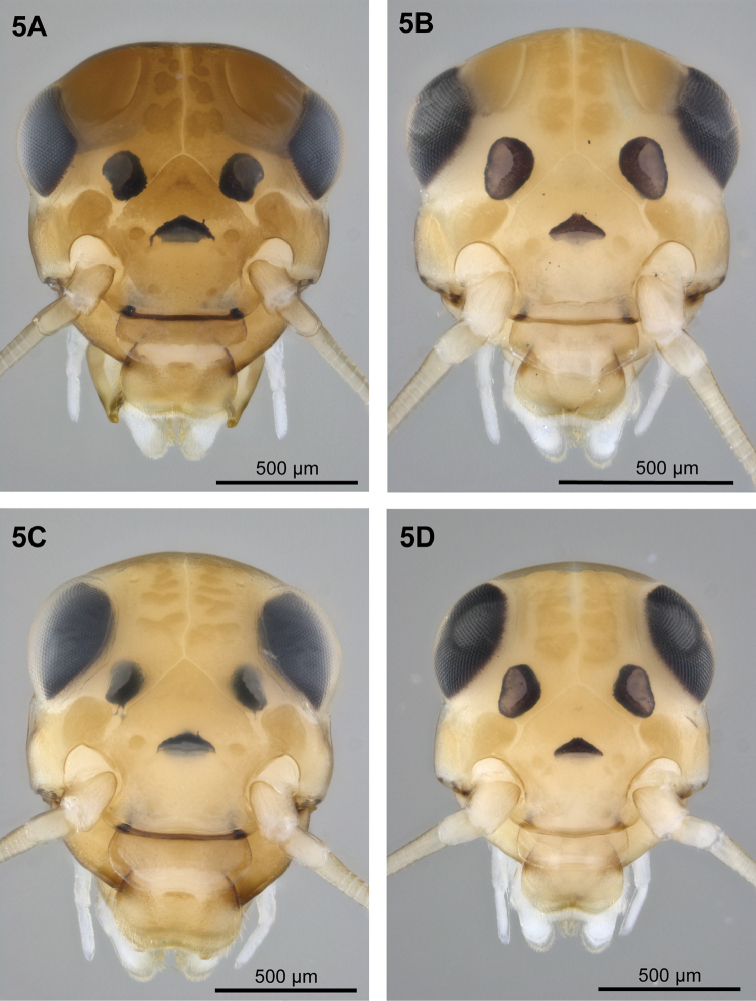
Colour pattern of Baetis (Baetis) cypronyx sp. n., larvae (**A, C** paratypes; material from Diplos River) and Baetis (Baetis) melanonyx (Pictet, 1843), larvae (**B, D** material from Germany): **5** head, dorsal view: **A–B** males **C–D** females.

**Figures 6–8.**
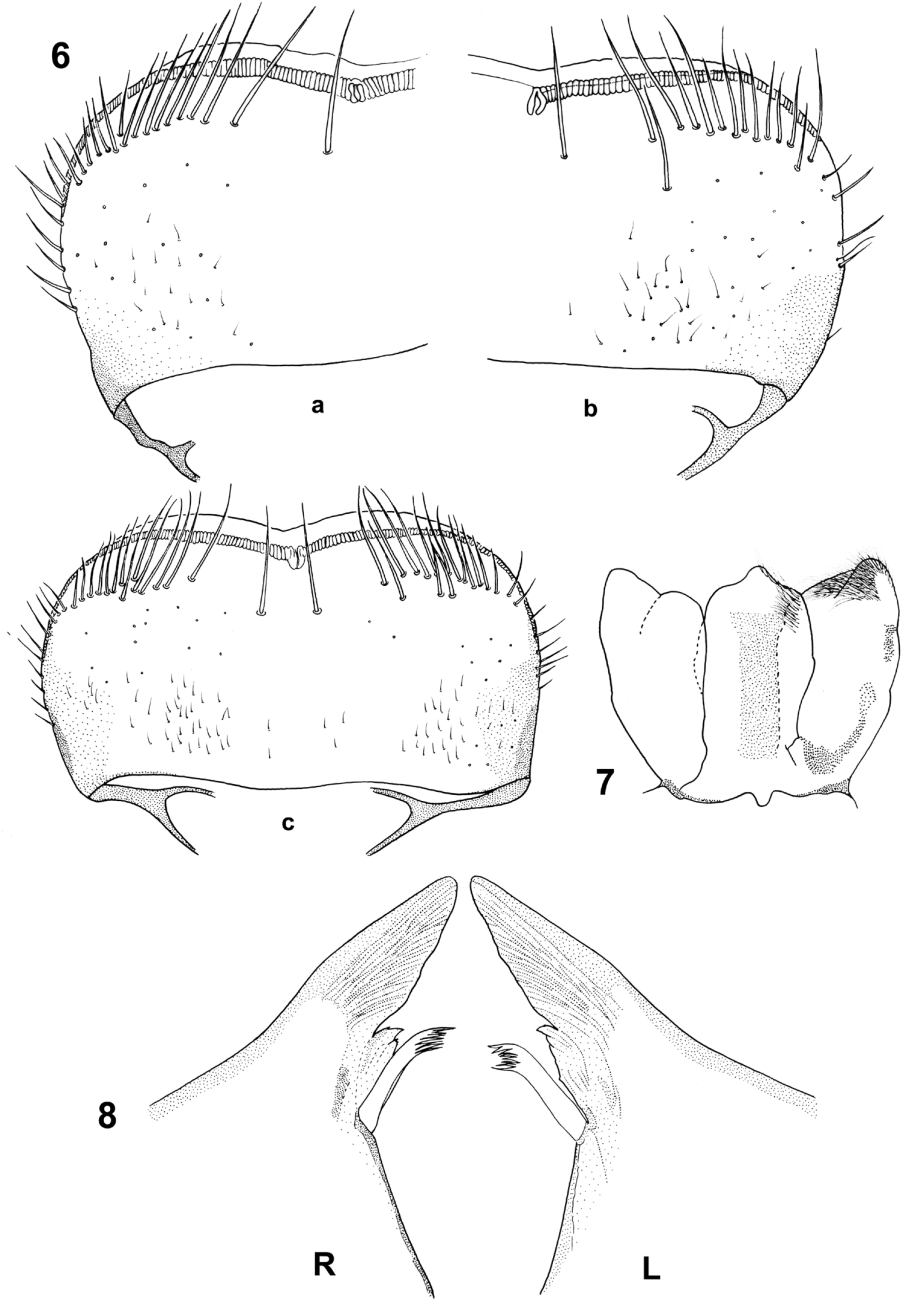
Baetis (Baetis) cypronyx sp. n., larva, paratypes, details of mouthparts: **6a−c** shape of labrum, dorsal view **7** hypopharynx **8** R: right mandible (incisors and prostheca), dorsal view; L: left mandibular (incisors and prostheca), dorsal view.

**Figures 9–12.**
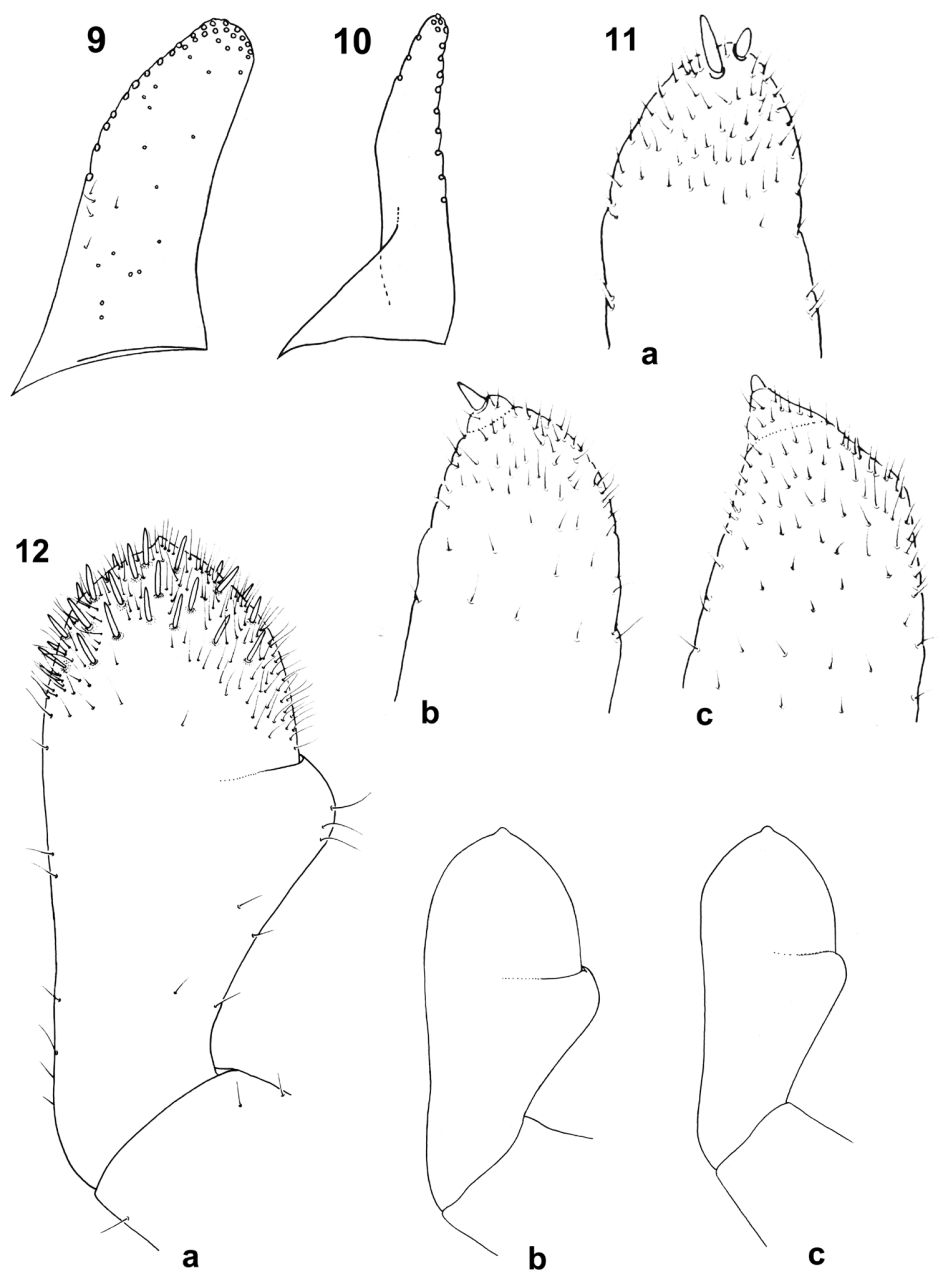
Baetis (Baetis) cypronyx sp. n., larva, details of mouthparts: **9** paraglossa, ventral view **10** glossa; ventral view **11a–c** apical part of maxillary palp, dorsal view **12a−c** shape of third segment of labial palps, ventral view.

**Figures 13–14.**
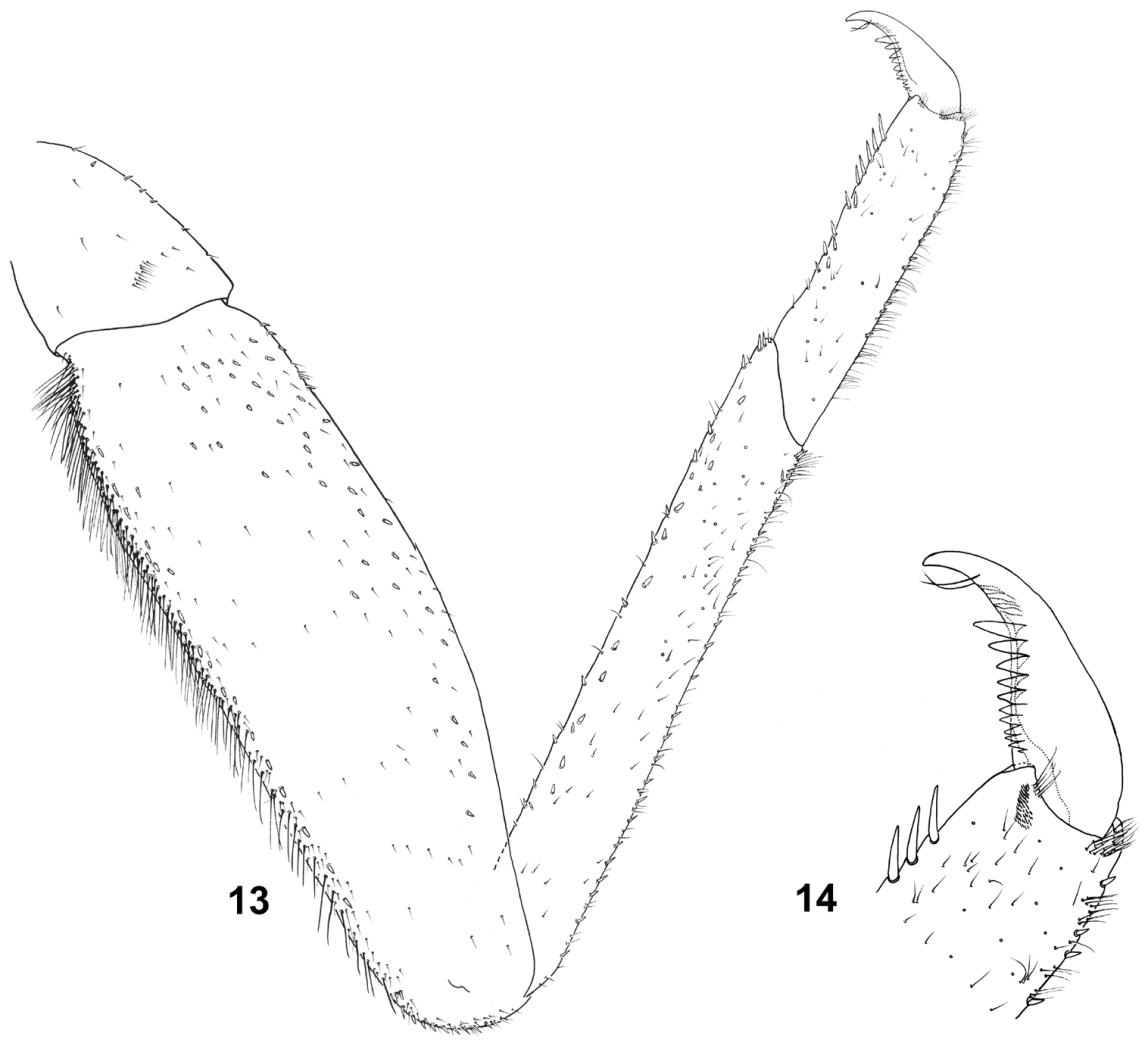
Baetis (Baetis) cypronyx sp. n., larva, paratype, hind leg: **13** general dorsal view **14** tarsal claw, dorsal view.

**Figure 15.**
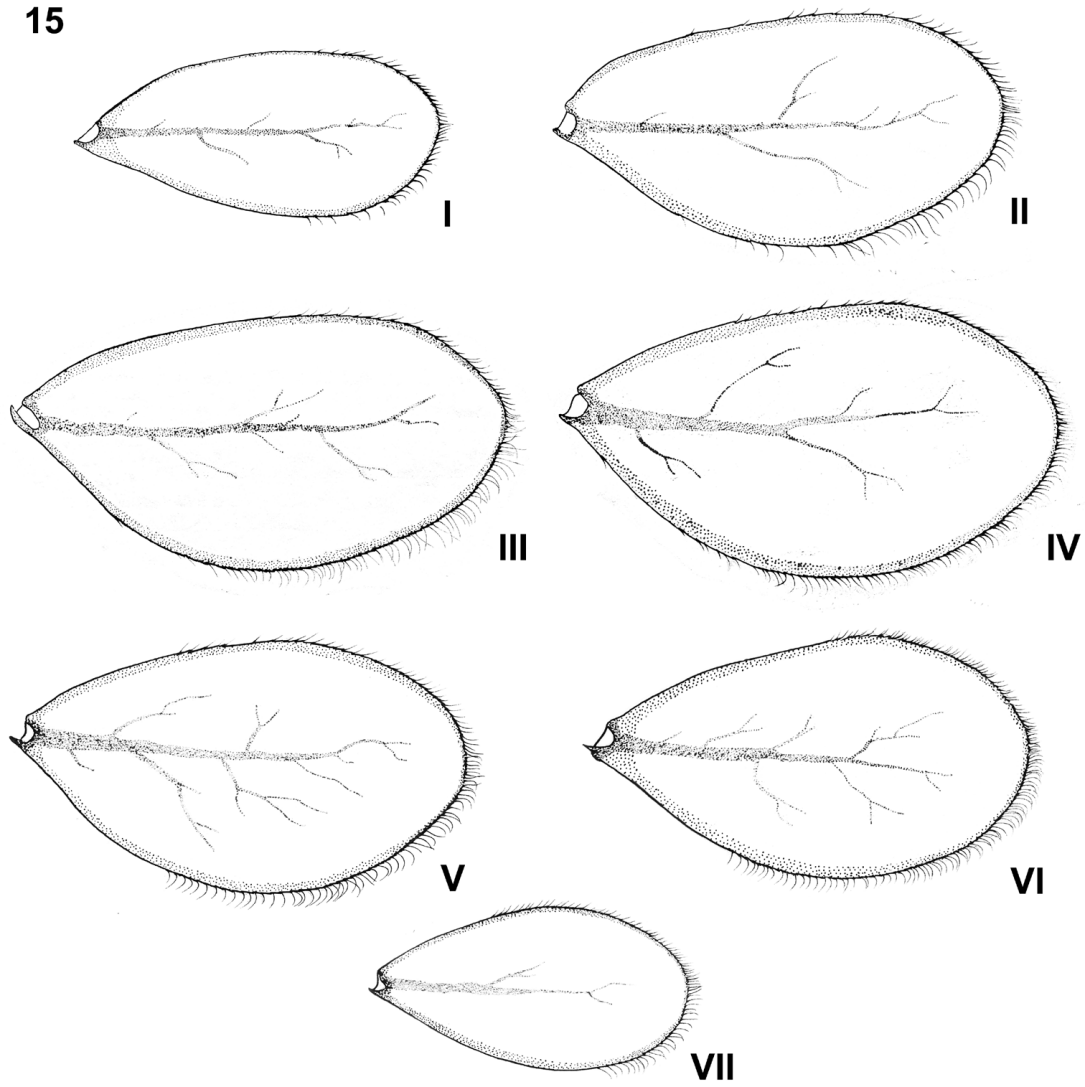
Baetis (Baetis) cypronyx sp. n., larva, paratype, gills. Roman numbers refer to the respective gill pairs.

**Figures 16–18.**
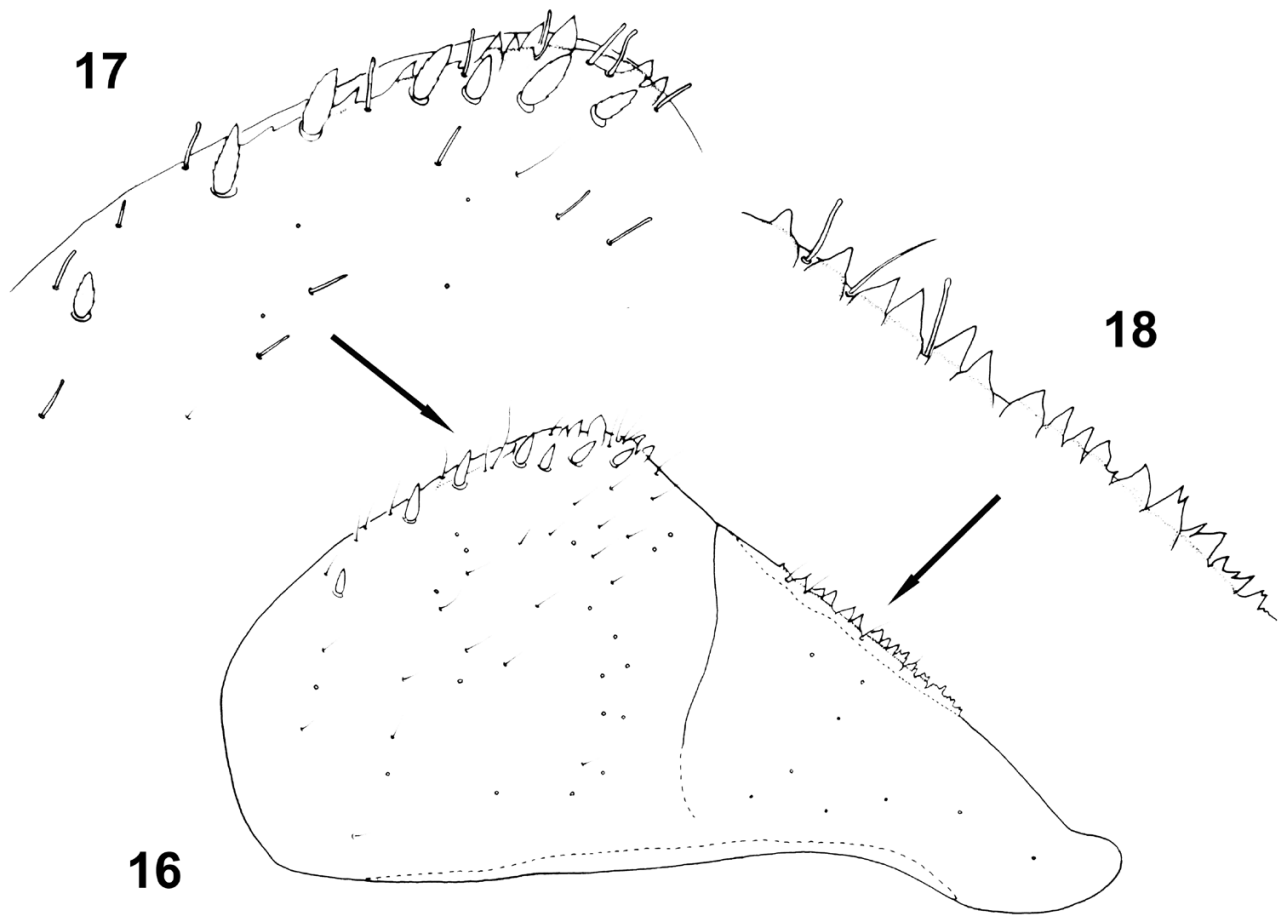
Baetis (Baetis) cypronyx sp. n., larva, paratype, details of paraproct: **16** paraproct, general ventral view **17** inner margin of paraproct plate, ventral view **18** spines of inner margin of cercotractor.

**Figures 19–22.**
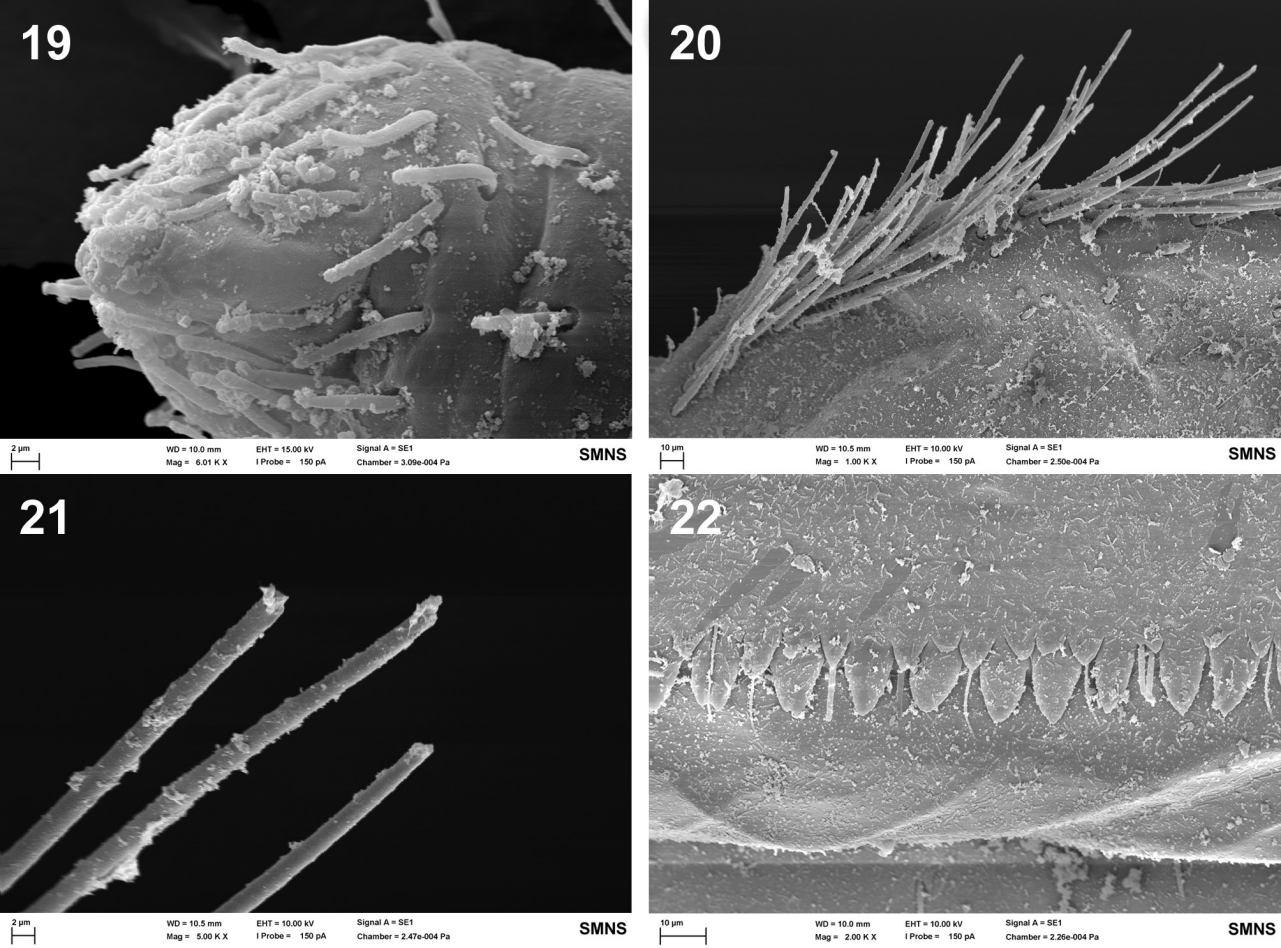
Baetis (Baetis) cypronyx sp. n., larva, SEM: **19** apical part of maxillary palp **20** outer margin of hind femur, proximally, dorsal view **21** apical part of long bristles of outer margin of femur, dorsal view **22** posterior margin of abdominal tergum V, dorsal view.

**Figures 23–24.**
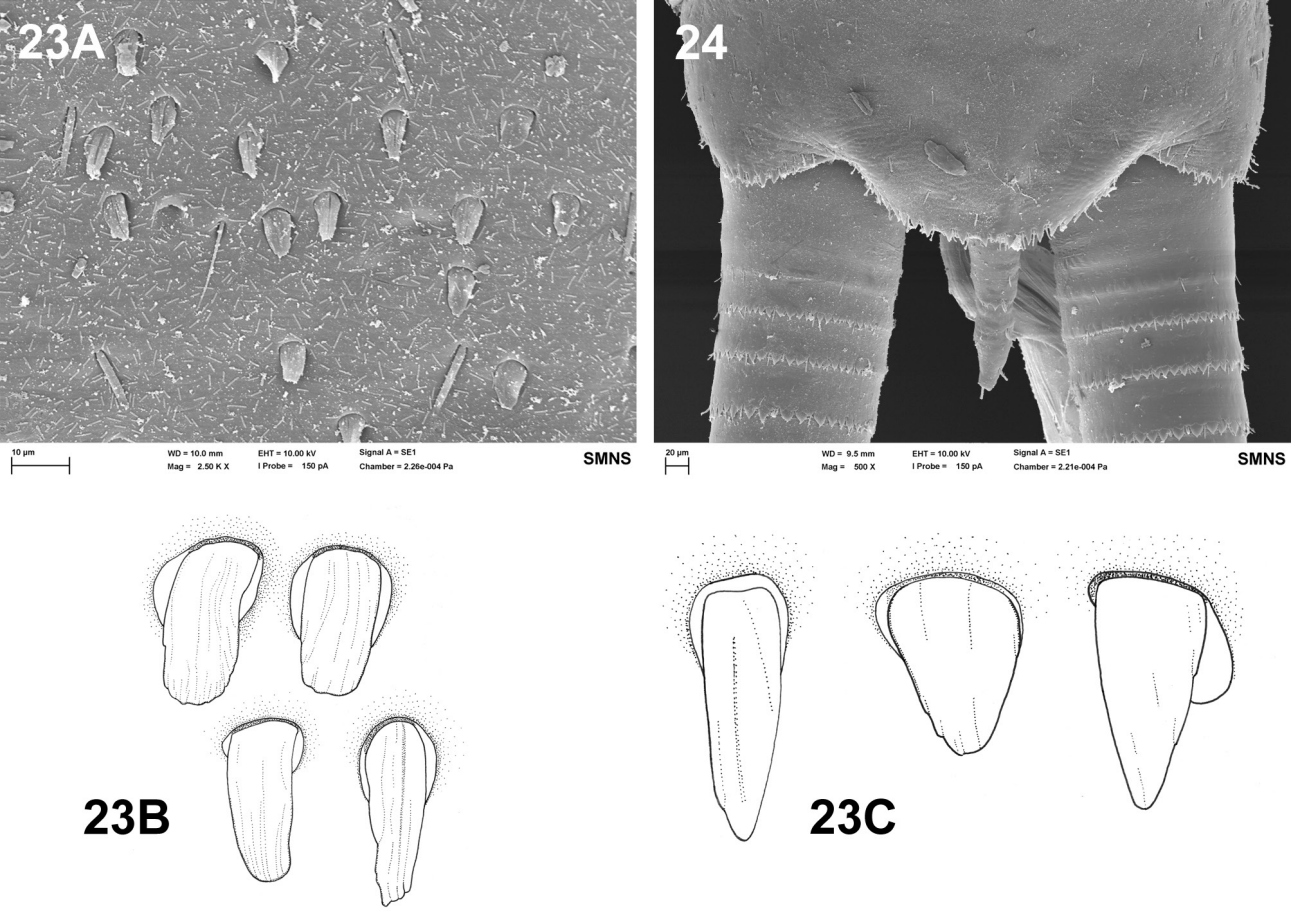
Baetis (Baetis) cypronyx sp. n., larva, SEM: **23A–C** surface of tergum VII: general dorsal view (**23A**); tongue-shaped scales [*SC-tg*] (**23B**); triangular scales [*SC-it*] (**23C**) **24** tergum X, dorsal view.

**Figures 25–28.**
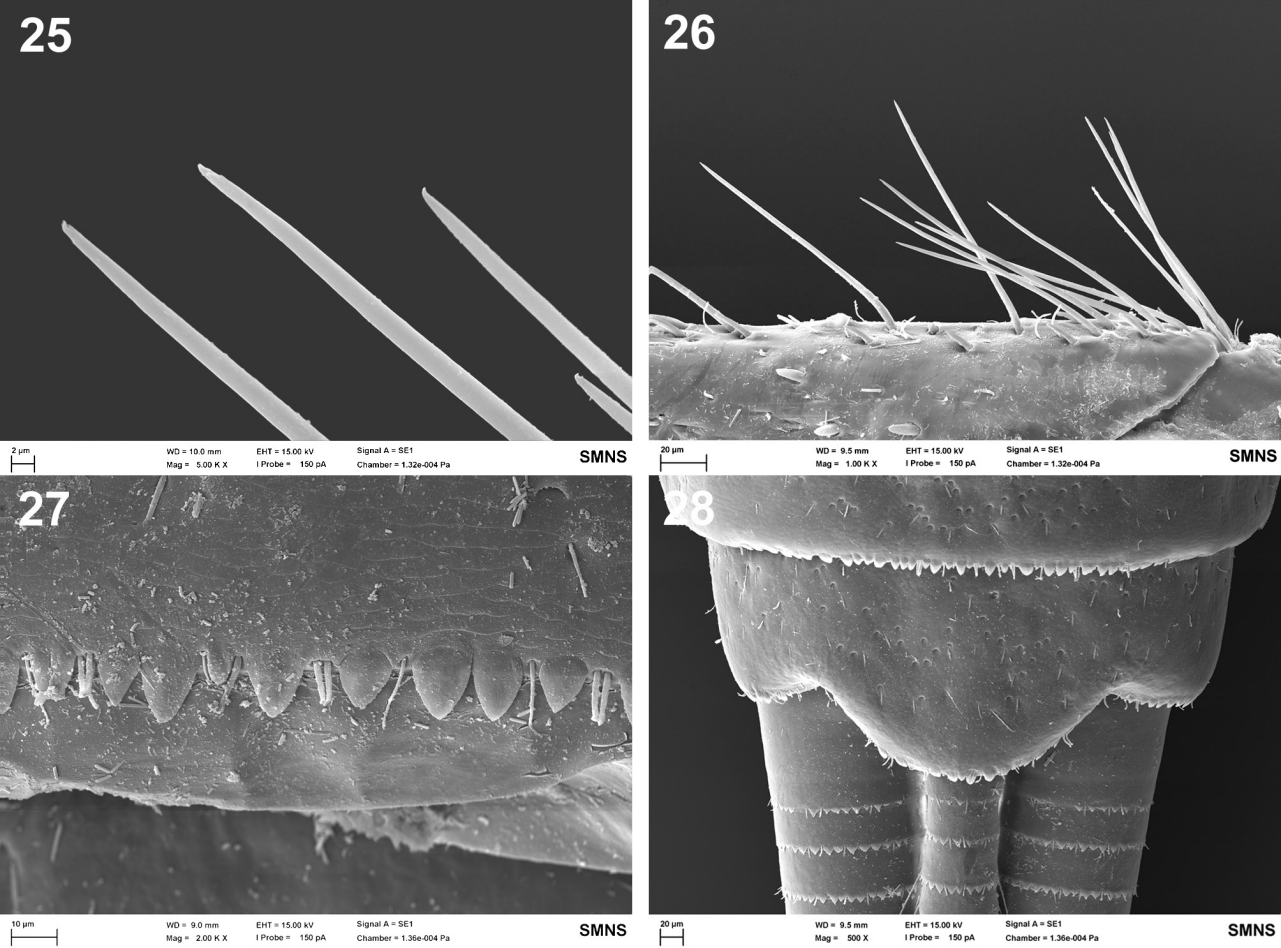
Baetis (Baetis) melanonyx (Pictet, 1843), Germany, larva, SEM: **25** apical part of long bristles of outer margin of femur, dorsal view **26** outer margin of hind femur, proximally, dorsal view **27** posterior margin of abdominal tergum V, dorsal view **28** tergum X, dorsal view.

**Figures 29–32.**
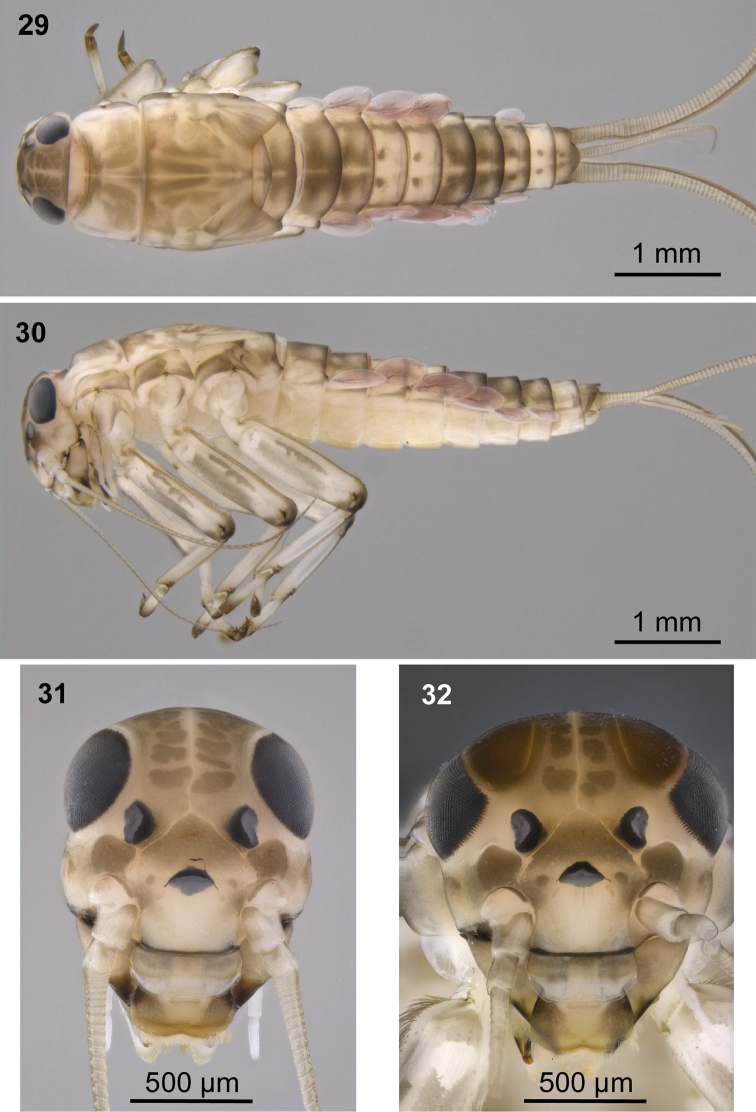
Colour pattern of *Baetis baroukianus* Thomas & Dia, 1984, larvae (material from Iran); male (**29, 30, 32**), female (**31**): **29** body, dorsal view **30** body, lateral view **31, 32** head, dorsal view.

## Taxonomy

### Baetis (Baetis) cypronyx sp. n.

Taxon classificationAnimaliaEphemeropteraBaetidae

http://zoobank.org/0B11F59C-97F2-42F2-AFD4-A68B6E3D3DCB

Figs 1–2 , 3–4 , 5 , 6–8 , 9–12 , 13–14 , 15 , 16–18 , 19–22 , 23–24

#### Type material.

***Holotype***: mature larva, CYPRUS, Limassol [Lemesos; Λεμεσός] District, Troodos [Τρόοδος] Mts., Kryos River [Κρύος ποταμός], Kalidonia Waterfalls, app. 1250 m a.s.l., N34 53.561 E32 52.043, 22.v.2004, leg. T. Soldán.

***Paratypes***: 75 larvae, the same date and place as holotype; 14 larvae, CYPRUS, Limassol [Lemesos; Λεμεσός] District, Troodos [Τρόοδος] Mts., Diplos River [Διπλός ποταμός], Chantara [Xantara] Waterfalls, near Trooditissa [Μοναστήρι Τροοδίτισσας] Monastery, app. 1300 m a.s.l., N34 54.429 E32 50.303, 23.v.2004, leg. T. Soldán;

4 larvae, ibid., Paphos District [Επαρχία πάφου], Gialia River [Γιαλιά], in the forest “Pochalantra”, app. 5 km upstream from Gialia [Γιαλιά] village, app. 400–410 m a.s.l., N35 04.364 E32 33.575, 12.xi.2005, leg. A. Buffagni;

10 larvae, United Nations Buffer Zone in Cyprus, Nicosia District [Επαρχία Λευκωσίας], upstream of Kargotis River [Καρκώτη], vicinity of Kakopetriya [Κακοπετριά] village, Mitro place, app. 150–200 m a.s.l., N34 59.012 E32 54.000, 22.iii.2006, leg. A. Buffagni;

2 larvae, ibid., Agios Nikolaos Lefkas [Άγιος Νικόλαος Λεύκας] village (abandoned), app. 100–120 m a.s.l., N35 5.280 E32 53.500, 24.iii.2006, leg. A. Buffagni.

#### Comparative material.

*Baetis baroukianus* Thomas & Dia, 1984: 1 male and 1 female mature larvae, LEBANON, Chouf District, type locality of *Baetis baroukianus*, branch of Salam (Râs el Mâ) spring near Harêt Jandal Municipality, app. 800 m a.s.l., 25.vii.1979, leg. Dia A. (see Thomas and Dia 1984: 10).

28 larvae (10 males, 18 females), IRAN [*new record*], Elburz Mts., Gilan Province, Rudbar County, Central District, unnamed brook in Divresh village, right tributary of Siah Rud River (SE upstream of Shirkooh village), app. 285 m a.s.l., N36 53.59 E49 35.06, 13.v.2016, leg. Bojková J., Soldán T. & J. Imanpour Namin, det. Sroka P.

2 larvae (1 male, 1 female), ibid, Fuman County, Sardar-e Jangal District, unnamed brook below of Masuleh City (right tributary of Rudkhan River), app. 710 m a.s.l., N37 09.42 E49 01.17, 22.v.2016, leg. Bojková J., Soldán T. & J. Imanpour Namin, det. Sroka P.

3 larvae (1 male, 2 females), ibid, Rudbar County, Central District, unnamed brook, left tributary of Sefīd-Rūd River, below Rostamabad City, app. 155 m a.s.l., N37 09.47 E49 00.17, 22.v.2016, leg. Bojková J., Soldán T. & J. Imanpour Namin, det. Sroka P.

*Baetis melanonyx* (Pictet, 1843): 30 larvae (7 larvae mounted with Liquide de Faure), Czech Republic, Ústí nad Labem district, Elbe river-basin, Divoká Orlice River, Líšnice village, 432 m a.s.l., 2.vii.1972, leg. T. Soldán (for details see Soldán 1978); 24 larvae (8 larvae mounted with Euparal), Germany: Baden-Württemberg, Boll, vor Tannegger Wasserfall, Wutach River, 623 m a.s.l., 03.vi.2008, leg. B. Frey. For other comparative material of *Baetis melanonyx* see Godunko (1999).

#### Diagnosis.

*Baetis cypronyx* sp. n. differs from all other representatives of the *Baetis alpinus* species-group by the following combination of larval characters (see Table 1): (**i**) labrum of distinctly oblong shape, nearly rectangular (Fig. 6a–c), (іі) outer mandibular incisor group distinctly fused, narrow and triangular (Fig. 8); (**iii**) segment 2 of maxillary palps usually with single seta, exceptionally with two stout apical setae (Figs 11a–c, 19); (**vi**) paraglossae with two irregular rows of long, stout bristles apically (Fig. 9); (**v**) segment 3 of labial palps not elongated, nearly symmetrical and evenly rounded (Fig. 12a–c); (**vi**) sternal protuberances on meso- and metathorax pointed apically; (**vii**) outer margin of femora with 2–3 rows of long, apically obtuse to bluntly pointed bristles proximally and centrally (Figs 13, 20, 21); (**viii**) irregular row of small submarginal spines on abdominal terga III–VIII (IX) (Fig. 22); (**ix**) surface of abdominal terga with few scales in sockets, scales triangular to tongue-shaped, not elongated, mostly lacking on tergum X (Figs 23, 24); (**x**) paraproct plate with bluntly pointed stout setae near to inner margin (Figs 16, 17); (**xi**) paracercus strongly reduced, 2–16 segmented (Fig. 24).

#### Description.

***Mature larva***: female body length: 7.5−8.0 mm, length of cerci: 9.0−11.5 mm; male body length: 6.0−8.0 mm; length of cerci: 7.0−10.0 mm; paracercus vestigial or strongly reduced.

*Cuticular coloration* (Figs 1–5). Due to ten to twelve years of material storage in ethanol, the herein described colour pattern might be slightly paler compared to fresh material.

General colour yellowish brown to brown. Head light brown with paler genae; clypeus light brown; frons with several small, isolated brown spots. Antennae light brown, flagellum paler than scape and pedicel.

Pronotum yellowish brown with two pairs of oblique brownish bands; mesonotum yellowish brown to brown, with longitudinal brown bands centrally, and several spots of the same colour centrally and laterally; metanotum brown with darker smudge centrally (Figs 1, 4). Lateral sides of thorax with brown pleurites (Fig. 2). Ventral side of thorax paler than dorsal side; sterna yellowish (Fig. 3). Legs pale. Femora yellowish brown with two darker, usually isolated longitudinal spots along outer margin; tibia light brown; base and apex of tarsi brown, darker than middle part; tarsal claw brown (Figs 2, 3).

Abdominal terga (Figs 1, 4) yellowish brown to brown; terga I–III (IV) and VI–VIII darker. Terga I–III (IV) brownish centrally, with broad pale area laterally; median brown spot on terga III and IV occasionally divided into two longitudinal spots; all terga with more or less well visible brownish band along anterior margin of segment; a pair of diffuse brownish maculae near posterior margin of terga V–VIII; a pair of brownish paramedian dots on terga II–X, terga III–VI occasionally with additional oblique streaks fused with paramedian dots and forming a diffuse brownish U-shaped pattern in anterior half of segment. Abdominal sterna with a pair of sublateral elongated spots. Cerci yellowish brown to brown, 3–5 first segments slightly darker.

*Hypodermal coloration*. Hypoderm without contrasting markings.

*Head*. Surface of clypeus and frons covered with solitary *FT*, *B*, and *Hr* setae. Larval turbinate eyes brown to intensively brown apically. Antennae slightly longer than 1/2 of body length. Scape and pedicel with solitary *FT* and *Hr*, and more abundant *B* setae only, without any particular cuticular ornamentation (e.g. corrugation/chagrin; see Bauernfeind and Soldán 2012), which is present in some representatives of the *Baetis alpinus* species-group and in the closely related *Baetis lutheri* and *Baetis pavidus* species-groups.

*Mouthparts*. Labrum (Figs 6a–c) distinctly wider than long, nearly rectangular, with width/length ratio 1.80–1.88; dorsal surface with 1 + 11–18 long submarginal setae, arranged in a single irregular row (occasionally 1–4 bristles form an additional weekly defined row); 6–8 smaller setae laterally on both margins; dorsal surface of labrum covered with sparsely arranged *B* and only a few *FT* setae grouped mainly posterolaterally; ventral side with 2–5 small pointed setae anterolaterally. Median incurvation of middle part of anterior margin of labrum clearly shallow and wide.

Outer mandibular incisor group narrow and triangular, distinctly fused; inner incisor group not prominent, with 3–4 small teeth (of which most distal tooth is the biggest), both groups separated by a shallow incision. Right and left prostheca of same size, nearly symmetrical, with 8–10 apical teeth (Fig. 8).

Maxillary palp two-segmented; segment 1 shorter than second segment; segment 2 asymmetrical apically, with pronounced tip (conical protuberance), and one distinct, stout seta; one additional stout seta occasionally near apex of segment 2; surface of both segments with *B* setae [uniporous sensillum basiconicum sensu Gaino and Rebora (2003: 449, figs 19−21)] which are clearly dense on distal part of segment 2 (Figs 11a–c, 19).

Hypopharynx relatively slender, anterior side laterodistally covered with fine, elongated setae along outer margins of lingua and superlinguae, lingua with prominent central lobe, superlinguae with marked hump (Fig. 7).

Labium with relatively slender glossae, slightly shorter than paraglossae (Figs 9, 10); glossae each with 8–10 stout bristles on inner margin, and 3–5 bristles on outer margin; 5–7 pairs of long, stout bristles form two irregular rows on tip of paraglossae; additionally 5–10 long bristles along outer margin and 3–5 medium sized setae on ventral side of paraglossae. Segment 2 of labial palp 1.30–1.42 longer than segment 3, covered only with sparse *B* and *Hr* setae; segment 3 not elongated, nearly symmetrical and evenly rounded, only slightly broader than long (width/length ratio 1.03–1.07); surface of segment 3 with 18–25 slender, pointed, stout setae [long and short hairs sensu Gaino and Rebora (2003)], and long *Hr* setae; quotient *q* changes from 0.76 to 0.88 (see Sroka et al. 2012, 29, 31: fig. 2) (Fig. 12a–c).

*Thorax*. Surface of pronotum with few *FT* and *Hr* setae only. Sternal protuberances on meso- and metathorax well visible, pointed apically, yellowish brown to brown.

Outer margin of femora with 2–3 rows of long bristles with obtuse to bluntly pointed tips proximally and centrally (Figs 13, 20, 21), and one row of shorter and stouter obtuse bristles distally; central part of outer margin of femora occasionally with long bristles arranged in 1–2 rows. Long marginal bristles alternating with submarginal *STSm-bp* setae and elongated *Hr* setae. Inner margin with 2–6 *STSs-bp* setae near to proximal end. Surface of femora with *STSs-bp* and *STSs-ov* setae and tiny setae [*Hr* and more abundant *FT* setae]. Outer and inner margins of tibiae with *STSm-p* and *STSm-bp* setae and short *Hr* setae; surface of tibia with *STSs-bp* to nearly *STSs-ov* setae; a group of long *Hr* setae near distal end of outer margin of tibia. Tarsi with 6–10 middle to elongated *STS-p* setae along the inner margin, and several *STSm-p* and/or *STSm-bp* setae on outer margin; both margins of tarsi covered with tiny *Hr* setae; surface of tarsi with a few *FT* and more abundant *Hr* setae, and small *STS-bp* setae. Tarsal claws not elongated, moderately hooked; with 10–11 teeth arranged in single row and two subapical tiny *Hr* setae (Figs 13, 14).

*Abdomen.* Posterior margin of terga with broad triangular spines of different size, bluntly pointed or occasionally pointed apically; broader spines along posterior margin of terga III–VIII; spines alternating with 1-3 tiny *B* and a single *Hr* setae. Irregular row of smaller submarginal spines on terga III–VIII (IX) (Fig. 22). Surface of terga with few, not elongated, tongue-shaped [*SC-tg*] to triangular [*SC-it*; bluntly pointed to rounded apically] scales, and their few sockets (mainly lacking on tergum X), concentrated on central part of segment (Figs 23, 24); solitary *Hr* and more abundant *FT* setae stretched over the whole surface of terga I–X. Posterior margin and surface of sterna without spines, stout setae or scales, with *B* and *Hr* setae only.

Paraproct plate as in Figs 16–18. Inner margin of paraproct with 8–12 spines of different size along apical half, alternating with tiny setae [solitary *FT* and more abundant *B* setae], and 2–8 (mainly 4–7) submarginal *STSm-bp* setae (Figs 16, 17); a single row of relatively small and stout spines along inner margin of cercotractor (for definition of cercotractor see Kluge 2004) (Fig. 18). Surface of paraproct covered with sparse *FT*, *B* and *Hr* setae and their bases only.

Tracheal gills whitish yellow to light brown, not elongated, broadly rounded apically (Fig. 15, I–VII); gills I and VII nearly symmetrical; gills II–VI asymmetrical; serrated margins of gills more or less well marked, with tiny *Hr* setae inserting in small, articulated bases; tracheation poorly visible.

Cerci as long as 1.20–1.32 of body length. Paracercus reduced to 2–16 segments (Fig. 24). Posterior margin of cercal and paracercal segments with row of broad, triangular spines, and uneven submarginal row of smaller spines. Length of paracercus of mature larvae apparently variable in different populations, as well as in specimens within each population. Paracercus in larvae from Cryos River (type locality) vestigial (evidently shorter than abdominal tergum X, consisting of approximately up to 5–7 segments, some segments at least partially fused, Fig. 1); paracercus in paratype larvae from Diplos River strongly reduced (but evidently longer than abdominal tergum X), only consisting of about 10 or more apparently separated or distinguishable segments; Fig. 4).

#### Male and female adults.

Unknown.

#### Etymology.

The specific epithet is a combination of the name of Cyprus, where the new species was found, and the specific epithet of the closely related species *Baetis melanonyx*.

## Discussion

### Affinities

*Baetis cypronyx* sp. n. can be undoubtedly attributed to the *Baetis alpinus* species-group as defined above based on larval body shape and presence of (**i**) numerous submarginal long setae on dorsal surface of labrum, (**ii**) triangular outer mandibular incisor group, (**iii**) 1–2 stout setae at tip of maxillary palp segment 2, (**iv**) conspicuous brownish pattern on pronotum (similar to that in *Baetis alpinus* (Pictet, 1843)) and well visible pair of dark spots on abdominal terga, (**v**) numerous long bristles on outer margin of femora, (**vi**) relatively large spines on posterior margin of terga, (**vii**) a pair of hair-like setae near tip of tarsal claw (see e.g. Müller-Liebenau 1969: 47; Jacob 2003: 67–68; Bauernfeind and Soldán 2012: 100–101).

The new species appears to be closely related to *Baetis melanonyx* known throughout Europe and to *Baetis baroukianus* Thomas & Dia, 1984 described from Lebanon. For the latter two species a separate subgenus Patites Thomas & Dia, 1999 was established based on larval and imaginal characters (Thomas and Dia 1999: 107; type species Baetis (Patites) baroukianus Thomas & Dia, 1984). On the other hand, Bauernfeind and Soldán (2012: 101) consider that the delimitation of taxa of *Baetis alpinus* species-group is rather difficult due to the high level of (probably clinal) variability combined with disjunctive area of many species. A separation of the *Baetis alpinus* species-group on genus or subgenus level is recently not considered to reflect phylogenetic lineages under the concept used for genera within *Baetinae* by these and other authors (e.g. Jacob 2003: 89; Bauernfeind and Soldán 2012: 101).

*Baetis cypronyx* sp. n., *Baetis baroukianus*, and *Baetis melanonyx* can be characterised by a distinctly fused, narrow and triangular outer mandibular incisor group; this character clearly distinguishes them from all other representatives of the *Baetis alpinus* species-group. Unfused teeth of outer mandibular incisors can be observed in *Baetis punicus* Thomas, Boumaiza & Soldán, 1983 and *Baetis berberus* Thomas, 1986 (Thomas et al. 1983: 108, fig. 3p; Thomas and Dia 1984: 8, fig. 4b; Peru and Thomas 2001: 77, fig. 2).

Differences between three above listed species can be observed in the arrangement of long setae on the dorsal surface of the labrum, i.e. *Baetis cypronyx* sp. n. with 1 + 11–18 long submarginal setae, in contrast to 1 + 14–21 long submarginal setae in *Baetis melanonyx*, and mainly 1 + 19–21 in *Baetis baroukianus* (Fig. 6a–c; Müller-Liebenau 1966: 70–78, figs 4–8; 1969: 62, fig. 27a; Thomas and Dia 1984: 8, fig. 2b). Additionally, in contrast to *Baetis melanonyx* with proximally narrowed labrum *Baetis cypronyx* sp. n. and *Baetis baroukianus* can also be characterized by a nearly rectangular labrum that is distinctly wider than long.

Two irregular rows of long, stout bristles can be observed on the tips of paraglossae in the new species, in contrast to 3 rows in *Baetis melanonyx* and 4–5 rows in *Baetis baroukianus* (see Table 1 and Fig. 9; Müller-Liebenau 1969: 62, fig. 27i; Thomas and Dia 1984: 8, fig. 6b).

Other differences concern the shape of labial palp segment 3, which is nearly symmetrical in *Baetis cypronyx* sp. n. and in *Baetis melanonyx* (quotient *q* from 0.76 to 0.94), in contrast to a markedly asymmetrical segment 3 in *Baetis baroukianus*, with *q* = 0.52–0.56 (Figs 12a–c; Thomas and Dia 1984: 9, figs 7a–c).

Thomas and Dia (1984: 7, 8, figs 1b, 1m) depicted the heads of the female larva of *Baetis baroukianus* and *Baetis melanonyx* in dorsal view, discussing the head width ratio for both species. For *Baetis baroukianus* was noted that its head is widest below the eyes between the genae, while in *Baetis melanonyx* the widest part was determined at eye level. According to Thomas and Dia (1984) the head width ratio for *Baetis baroukianus* / *Baetis melanonyx* below the eyes is 1.59 (with maximal value 1.46). However, in larvae of *Baetis baroukianus* from Iran that we examined, the width of head both at eye level and below eyes is nearly equal in both sexes, respectively (Figs 31, 32).

In contrast, female larvae of *Baetis cypronyx* sp. n. and *Baetis melanonyx* both have their maximal width of head at the level of eyes; the head width however is only slightly smaller at genal level below the eyes in both species (Fig. 5C, D). Similar proportions also apply for male larvae of the latter two species (Fig. 5A, B). The larval head width ratio for *Baetis cypronyx* sp. n. / *Baetis melanonyx* below the eyes at genal level is 1.05–1.15 in females, and 1.15–1.20 in males; at eye level the ratio is 1.00–1.05 in females, and 1.14–1.20 in males.

*Baetis melanonyx* and *Baetis baroukianus* markedly differ from the new species by their arrangement of setation at the outer margin of femora. There is only a single row of acutely pointed long bristles proximally and centrally, alternating with *STSe-bp* (in *Baetis melanonyx*) and *STSm-bp* (in *Baetis baroukianus*) submarginal setae, in contrast to *Baetis cypronyx* sp. n. that features 2–3 rows of bluntly pointed long bristles centrally and a group of *STSm-bp* submarginal setae (Figs 13, 20, 21). This character has been recently used for delimitation of two distinct evolutionary units of *Baetis alpinus* within the Central Alps (Leys et al. 2016), and much earlier for delimitation of *Baetis alpinus* and *Baetis melanonyx* (Figs 25, 26; Müller-Liebenau 1969; Godunko 1999: 26, fig. 3C).

The new species also clearly differs from *Baetis melanonyx* and *Baetis baroukianus* in the sternal protuberances near the coxae on meso- and metathorax that are pointed apically in the former species, in contrast to rounded apically protuberances in both latter species (Table 1).

Abdominal terga of *Baetis melanonyx* and *Baetis baroukianus* (including tergum X) are covered by numerous scale sockets, in contrast to only a few scales on terga of *Baetis cypronyx* sp. n., where scales and their sockets are missing on tergum X (see Figs 23, 24 for *Baetis cypronyx* sp. n.; Godunko 1999: 26, fig. 3D, and our Fig. 28 for *Baetis melanonyx* [the same for *Baetis baroukianus*]; Table 1); the shape of scales is similar in all three discussed species. The shape of marginal spines along the posterior margin of abdominal terga in all three species is generally similar, but the new species can be markedly recognized by the presence of not shortened stout spines and additional, submarginal, irregular row of smaller spines on terga III–VIII (IX) (see Fig. 22 for the new species in contrast to Fig. 27 for *Baetis melanonyx*); in *Baetis baroukianus* the single row of shortened stout spines is figured by Thomas and Dia (1984: 9, fig. 9). Marginal large spines alternating with *Hr* setae and with 1–3 setae of sensillum basiconicum type can be recognised in the new species (similarly to *Baetis alpinus*), in contrast to *Baetis melanonyx* and *Baetis baroukianus* showing a group of 1–5 setae.

Additional differences between the new species and the previously described *Baetis baroukianus* and *Baetis melanonyx* can be recognized in the colour pattern of abdominal terga. Thomas and Dia (1984: 10) noted similar colour patterns in *Baetis baroukianus*, *Baetis punicus*, and *Baetis alpinus*. Bauernfeind and Soldán (2012: 102, 106) discussed the presence of well pigmented paramedian dots and streaks or a mediolongitudinal strip on terga I (II)–IX (X) within all three species (Fig. 29). A similar pattern is described for *Baetis melanonyx*, but usually with terga IV, V and IX paler centrally (Müller-Liebenau 1966: 74–75, figs 6, 7; 1969: 52–53, 63–64, figs 19, 204).

Inner margin of paraproct plate of *Baetis cypronyx* sp. n. and *Baetis melanonyx* with more or less similar number of marginal spines (see Table 1), in contrast to *Baetis baroukianus* with not more than four spines only. Other differences between species discussed above can be recognized in the number and shape of submarginal stout setae, i.e. 2–8 *STSs-bp* and *STSm-bp* setae in *Baetis cypronyx* sp. n., in contrast to 8–12 in *Baetis melanonyx* and up to 7 *STSs-ov* and *STSm-ov* setae in *Baetis baroukianus*.

Clearly visible differences between these species can be also recognized in the shape and length of paracercus, i.e. strongly reduced in *Baetis cypronyx* sp. n., with 2–16 segments; shortened or well-developed in *Baetis baroukianus* (from 15 segments to 1/2–2/3 of cerci length); well developed in *Baetis melanonyx*, as long as 1/2–2/3 of cerci length (Figs 3, 4, 24, 28; Table 1).

Other differences between the closely related species *Baetis cypronyx* sp. n. *Baetis baroukianus* and *Baetis melanonyx* are summarized in Table 1.

Thomas and Gazagnes (1984) described *Baetis cyrneus* Thomas & Gazagnes, 1984 from Corsica and placed this species in the *Baetis alpinus* species-group. *Baetis cyrneus* most probably also is an endemite of the Mediterranean islands (see below). It differs from *Baetis cypronyx* sp. n. by the arrangement of mouthparts, especially by the shape and setation of mandible with both groups of incisors well developed, segment 2 of maxillary palps with two regular stout setae apically, and by the elongated shape of labial palp segment 3. Additional differences can be observed in the paraproct plate, with numerous scale sockets on its surface, and in the length of paracercus with 10–25 segments.

### Biological notes

**Figures 33–35.**
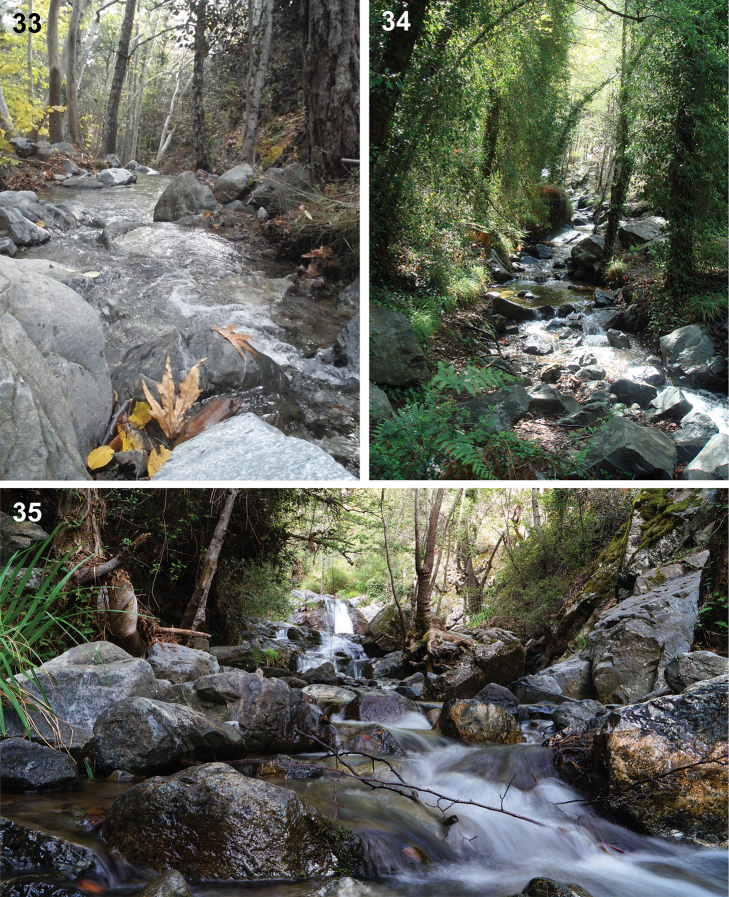
Localities of Baetis (Baetis) cypronyx sp. n.: **33** Kryos River [Κρύος ποταμός], app. 1270 m a.s.l., near type locality (photo by Zsuzsa Miskolci, Budapest, Hungary) **34** ibid., app. 1285 m a.s.l. (photo by Philp J Stoate, Somerset, England) **35** Diplos River [Διπλός ποταμός], Chantara [Xantara] Waterfalls, app. 1100 m a.s.l., locality of *Baetis cypronyx* sp. n. (photo by Alexandros Constantinides, Cyprus)

Larvae of *Baetis cypronyx* sp. n. were found solely on stony substrates (lithal) at depths of 5–40 cm (see also Soldán and Godunko 2008), preferably in stream sections with moderate to fast current (velocity approximately 20–50 cm/s^-1^) (Figs 33–35). The macroinvertebrate taxocene of both localities included several mayfly taxa, viz. *Baetis irenkae*, Baetis (Baetis) cf. muticus (Linnaeus, 1758), Epeorus (Ironopsis) sp., and *Electrogena* sp. Flight period probably from May and during first half of summer months, since several nymphs ready to emerge were collected together with younger larvae.

### Notes on distribution

As well as *Baetis irenkae*, a new species so far known only from several localities in Cyprus (type locality at Kryos River within Kalidonian Waterfalls, and another one locality at Diplos River within Chantara Waterfalls), and thus might be considered presently as endemic to this island (Table 2).

**Table 2.** Checklist of Baetidae in the Mediterranean islands (islands listed from west to east). Abbreviations and symbols: SP – Spain; IT – Italy; FR – France; MT – Malta; GR – Greece; CY – Cyprus. ● – previous records on occurrence of the species confirmed; ○ – occurrence based on our unpublished data; * − data on distribution and / or proper species identification require to be confirmed or clarified.

No. of species	Species / Mediterranean island Comments in [] see below	Baleares (SP)	Sardinia (IT)	Corsica (FR)	Elba (IT)	Sicily (IT)	Malta (MT)	Gozo (MT)	Crete (GR)	Tasos (GR)	Lesbos (GR)	Astypalea (GR)	Kos (GR)	Karpathos (GR)	Tilos (GR)	Rhodos (GR)	Cyprus (CY)
Genus *Baetis* Leach, 1815 Subgenus Acentrella Bengtsson, 1912																	
1	Baetis (Acentrella) sinaicus (Bogoescu, 1931)^[1]^					●											
Subgenus Baetis Leach, 1815 *Baetis alpinus* species-group																	
2	Baetis (Baetis) cypronyx **sp. n.**																●
3	Baetis (Baetis) cyrneus Thomas & Gazagnes, 1984^[2]^		●*	●		●*											
4	Baetis (Baetis) melanonyx (Pictet, 1843)^[3]^					●											
*Baetis buceratus* species-group																	
5	Baetis (Baetis) buceratus Eaton, 1870^[4]^		●	●		●											
6	Baetis (Baetis) zdenkae Soldán & Godunko, 2009^[5]^															●	
*Baetis lutheri* species-group																	
7	Baetis (Baetis) lutheri Müller-Liebenau, 1967^[6]^					●							●*				
8	Baetis (Baetis) mirkae Soldán & Godunko, 2008^[7]^															●	●
*Baetis pavidus* species-group																	
9	Baetis (Baetis) pavidus Grandi, 1951^[8]^					●											
*Baetis vernus* species-group																	
10	Baetis (Baetis) vernus Curtis, 1834^[9]^			●*													
*Baetis fuscatus* species-group																	
11	Baetis (Baetis) fuscatus (Linnaeus, 1761)^[10]^		●	●		●			●*								
Subgenus Nigrobaetis Novikova & Kluge, 1987																	
12	Baetis (Nigrobaetis) albinatii Sartori & Thomas, 1989^[11, 12]^			●													
13	Baetis (Nigrobaetis) digitatus Bengtsson, 1912^[13]^												●*			●*	
14	Baetis (Nigrobaetis) muticus (Linnaeus, 1758)^[14]^		●*	●*		●*											
15	Baetis (Nigrobaetis) cf. muticus (Linnaeus, 1758)																○*
16	Baetis (Nigrobaetis) cf. navasi (Müller-Liebenau, 1974)^[15]^								●*								
17	Baetis (Nigrobaetis) niger (Linnaeus, 1761)^[16]^			●*													
Subgenus Rhodobaetis Jacob, 2003																	
18	Baetis (Rhodobaetis) ingridae Thomas & Soldán, 1987^[17]^			●*													
19	Baetis (Rhodobaetis) irenkae Soldán & Godunko, 2008^[18]^																●
20	Baetis (Rhodobaetis) rhodani (Pictet, 1843)^[19]^	●	●*	●*		●*			○*	●			●*	●*			●*
21	Baetis (Rhodobaetis) cf. rhodani (Pictet, 1843)^[19]^		●*	●*	●*											○*	
Genus *Centroptilum* Eaton, 1869																	
22	*Centroptilum luteolum* (Müller, 1776)^[20]^		●	●		●				●			●	●			
Genus *Cloeon* Leach, 1815 Subgenus Cloeon Leach, 1815																	
23	Cloeon (Cloeon) cognatum Stephens, 1836^[21]^	●*	●*			●*											
24	Cloeon (Cloeon) dipterum (Linnaeus, 1761)^[22]^	●	●	●		●	○*	○*	○*	●		●	●	●	●		○
25	Cloeon (Cloeon) inscriptum Bengtsson, 1917^[23]^	●*															
26	Cloeon (Cloeon) rabaudi (Verrier, 1949)^[24]^			●*													
Subgenus Similicloeon Kluge & Novikova, 1992																	
27	Cloeon (Similicloeon) praetextum Bengtsson, 1914^[25]^	●*															
28	Cloeon (Similicloeon) schoenemundi Bengtsson, 1936^[26]^	●*															
29	Cloeon (Similicloeon) simile Eaton, 1870^[27]^	●	●	●		●				●							
Genus *Procloeon* Bengtsson, 1915 Subgenus Procloeon Bengtsson, 1915																	
30	Procloeon (Procloeon) bifidum (Bengtsson, 1912)^[28]^		●		●	●											
Subgenus Pseudocentroptilum Bogoescu, 1947																	
31	Procloeon (Pseudocentroptilum) fascicaudale (Sowa, 1985)^[29]^															●	
32	Procloeon (Pseudocentroptilum) pulchrum (Eaton, 1885)^[30]^					●*											
33	Procloeon (Pseudocentroptilum) unguiculatum (Tshernova, 1941)^[31]^										●						

1 Recorded by Belfiore (1983: 57) for the first time; recent data on its distribution summarised by Belfiore and D’Antonio (1991: 260), Belfiore et al. (1991: 32) and Buffagni et al. (2003: 281).

2 Described by Thomas and Gazagnes (1984: 199) from Corsica. According to Bauernfeind and Soldán (2012: 105) only known from a few localities in terra typica (see also OPIE-benthos data). Nevertheless, Belfiore (1988), Belfiore and D’Antonio (1991), and Buffagni et al. (2003) report *Baetis cyrneus* also from the Toscana Region and some Mediterranean islands, i.e. Sicily and Sardinia. DNA barcoding (Gattolliat et al. 2015) however revealed that specimens determined as *Baetis cyrneus* represent four different cryptic species occurring in Corsica and Sardinia. So far no morphological differences have been determined for these putative species. The high intra-specific genetic distance in *Baetis cyrneus* recently detected by Cardoni et al. (2015) for populations from Corsica and Sardinia also point to cryptic variation.

3 Recorded by Belfiore and D’Antonio (1991) and Belfiore et al. (1991) for the first time.

4 Recorded by Belfiore and Gaino (1988: 77) for the first time in Sardinia and later also listed from Sicily (Belfiore and D’Antonio 1991; Buffagni et al. 2003); for Corsica based on OPIE-benthos data.

5 So far known only from type locality and a single additional locality in Rhodos (Soldán and Godunko 2009: 7−8), considered endemic to the island.

6 The record from Kos by Belfiore (1990: 266) probably belongs to or other, still undescribed species of the *Baetis lutheri* species-group.

7 Considered as probably East Mediterranean (Pontomediterranean) species by Bauernfeind and Soldán (2012: 124); so far known from three localities in Cyprus and from a single locality in Rhodos (Soldán and Godunko 2008: 95).

8 Recorded by Belfiore (1983) for the first time; recent data on distribution summarized by Belfiore and D’Antonio (1991: 260), Belfiore et al. (1991: 32) and Buffagni et al. (2003: 281).

9 Most probably missing on Mediterranean islands (Bauernfeind and Soldán 2012: 136). The record for Corsica by Sartori and Thomas (1989: 131) based on earlier data by Verrier (1954: 282 [sub. *Baetis* type *vernus*]; 284 [sub. *Baetis vernus*]) needs to be verified.

10 Reported from Corsica by Esben-Petersen (1912: 351, 1913: 22) [sub. *Baëtis binoculatus* Linn.]; Kimmins (1930: 186) and Lestage (1922: 275; citation of M. Esben Petersen data) [sub. *Baetis binoculatus* L.] (see also OPIE-benthos data). Verrier (1954: 284; 1956: 95) reported [sub. *Baetis bioculatus* L.] and [sub. *Baetis*, type *bioculatus* L.] from four localities in Crete, but conspecifity with *Baetis fuscatus* needs to be verified. For Sicily and Sardinia see summarized data in Belfiore (1983), Belfiore and D’Antonio (1991), Belfiore et al. (1991) and Buffagni et al. (2003). Recent data on DNA barcoding by Cardoni et al. (2015) based on Sardinian material.

11 The species can be considered endemic to Corsica. This conclusion is confirmed by recent DNA barcoding (Gattolliat et al. 2015; Cardoni et al. 2015). All previous records of *Baetis muticus* from Corsica refer to *Baetis albinatii* (see e.g. Hagen 1864: 39 [sub. *Cloe Pumila* Burm.]; Jakobson and Bianki 1905: 875 and Klapálek 1917: 193 [sub. *Baëtis pumilus* (Burm.)]; Esben Petersen 1913: 22 [sub. *Baëtis pumilus* Burm.]; Kimmins 1930: 186 [sub. *Baetis pumilus* Burm.] (citation of previous authors); Belfiore and D’Antonio 1991: 260 [sub. *Baetis muticus* (*L.*)]; see also OPIE-benthos data). Three species reported for Corsica by Hagen (1864: 38) within the genus *Baetis* (orig. *Baetis* Leach.) belong to the genera *Ecdyonurus* Eaton, 1868 and *Electrogena* Zurwerra & Tomka, 1985.

12 Sartori and Thomas (1991: 224) used the specimens from the type series of *Baetis albinatii* also to specify distinguishing characters of representatives of the *Baetis muticus* species-group.

13 Two records of this species from the islands Kos (Belfiore 1990: 266) and Rhodos (Soldán and Godunko 2009: 9) belong to hitherto undescribed species.

14 Reported by Grandi (1960), Belfiore et al. (1991), Belfiore and D’Antonio (1991) and Buffagni et al. (2003) from Sicily and Sardinia. The record from Corsica (Belfiore and D’Antonio 1991) in fact refers to *Baetis albinatii* (see above). The presence of new undescribed endemic species in Sardinia is confirmed based of DNA barcoding by Gattolliat et al. (2015).

15 Reported by Bauernfeind (2003: 100) based on a single male imago and two subimagines, with remarks on similarities to *Baetis navasi* Müller-Liebenau, 1974, but with some differences from continental *Baetis muticus*.

16 Reported by Esben Petersen (1912) and Kimmins (1930; citation follows data by M. Esben-Petersen); the record by Lestage (1922) also follows data by M. Esben-Petersen; recently reported from Corsica based on published data (see OPIE-benthos). Bauernfeind and Soldán (2012: 154) consider this record questionable.

17 Most probably endemic to Corsica (Thomas and Soldán 1987: 23; Bauernfeind and Soldán 2012: 167). All previous reports of *Baetis rhodani* from Corsica (see e.g. Hagen 1864: 39 [sub. *Cloe Rhodani* ? Pictet]; Jakobson and Bianki 1905: 875 [sub. *Baetis rhodani* (Pict.)]; Lestage 1922: 275 [sub. *Baetis Rhodani* Pict.]; Kimmins 1930: 186 [sub. ? *Baetis rhodani* Pict.] (cited following Hagen 1864); Belfiore and D’Antonio 1991: 260 [sub. *Baetis rhodani* (Pictet)]) most probably belongs to *Baetis ingridae* (see also OPIE-benthos data). At least a part of the material marked as “*Baetis* sp.” by Verrier (1954) from rivers Bevinco, Golo, Restonica, Vecchio, Travo and Rizzanèse in Corsica also belongs to *Baetis ingridae* Thomas & Soldán, 1987). Recent investigation of DNA barcodes of Corsican mayflies by Gattolliat et al. (2015) clearly showed that it is not possible to assign the separate lineage of this species to a proposed insular Corso-Sardinian lineages; additional investigation of type material is urgently needed to clarify the systematic status of these questionable taxa.

18 So far only known from Cyprus; probably endemic to the island (Soldán and Godunko 2008: 91).

19 The records from Corsica might in fact belong to *Baetis ingridae* and/or new undescribed species (see above). Taxonomical status of larval material reported by Verrier (1956: 95) from Crete [sub. *Baetis*, type *gemellus* Etn.] needs to be clarified. *Baetis rhodani* was formally listed for Sardinia by Buffagni et al. (2003). Taxonomical status of material from the Mediterranean islands attributed to “*Baetis rhodani*” remains unclear, since the existence of series of cryptic species among European populations is confirmed by molecular taxonomy (see Williams et al. 2006; Lucentini et al. 2011; Gattolliat et al. 2015). In Italy, 11 potential cryptic species have been recognized, one of these cryptic species clearly has a restricted geographical range within Sicily only (see the position of cryptic species G9 in Lucentini et al. 2011). Gattolliat et al. (2015) documented the existence of two separate insular clades (three clear lineages) for Corso-Sardinian material of Baetis gr. rhodani. Finally, Bisconti et al. (2016) reported about occurrence of three distinct and deeply divergent species within the “*Baetis rhodani* species group” in the north-western Mediterranean islands (Sicilia, Corsica and, Elba) based on DNA analysis.

20 Numerous records from the Mediterranean islands. The record for Tasos was published by Russev (1959: 272) [sub. *Gentroptilum* (sic!) *luteolum* Müller]; first record for Sicily by Grandi (1966: 327); first record for Corsica most probably by Verrier (1954) [sub. *Centroptilum* sp.]; the records for Kos and Karpathos by Belfiore (1990: 267). Recent data on distribution in Sicily, Sardinia and Corsica are summarized by Belfiore and D’Antonio (1991), Belfiore et al. (1991) and Buffagni et al. (2003).

21 Listed for Sicily and Sardinia by Belfiore and Gaino (1988) and Belfiore and D’Antonio (1991), but absent in the tabular list of Italian species summarized by Buffagni et al. (2003). Original record from Sicily of Belfiore (1983) concerns *Cloeon dipterum* (see Belfiore et al. 1991). The original record from the Balearic Islands needs to be confirmed (Alba-Tercedor and Jáimez-Cuéllar 2003: 92). *Species inquirenda* according to Bauernfeind and Soldán (2012: 189).

22 Numerous records from Mediterranean islands. Russev (1959: 272) recorded this species from the island of Tasos [sub. *Cloëon rufulum* Eaton]. We collected this species in Malta for the first time (previously unpublished data). However, this findings were generally mentioned in a tabular summary on the distribution of European mayflies (Bauernfeind and Soldán 2012: 639), actual data on respective localities have never been published. These are as follows: Island of Malta: Wied il-Qleja [brook], small artificial reservoirs called Chadwick Lakes, about 15 km west of the town Intarfa, about 110 m a.s.l., N35 89.100 E14 38.580, 238 larvae, 85 males, 15 females, 8 subimagoes, May 12, 2010; Island of Gozo, unnamed brook, about 2 km south of the town Malsarforn, about 10 m a.s.l., N36 07.014 E14 26.010, 22 larvae May 15, 2010 (all material leg. T. Soldán).

23 The record from the Balearic Islands needs to be confirmed (Alba-Tercedor and Jáimez-Cuéllar 2003). *Species inquirenda* according to Bauernfeind and Soldán (2012: 191).

24 Verrier (1954: 284) reported larvae from Lake Nino (Corsica) [sub. *Procloeon Rabaudi* Verrier]. According to the online portal Fauna Europaea [http://www.faunaeur.org/] junior subjective synonym of *Cloeon simile*. *Species inquirenda* according to Bauernfeind and Soldán (2012: 194). Taxonomical status and presence in Corsica needs to be clarified.

25 The record from the Balearic Islands needs to be confirmed (Alba-Tercedor and Jáimez-Cuéllar 2003). Most probably part of material belongs to *Cloeon simile. Species inquirenda* according to Bauernfeind and Soldán (2012: 199).

26 The record from the Balearic Islands needs to be confirmed (Alba-Tercedor and Jáimez-Cuéllar 2003). Most probably part of material belongs to *Cloeon simile. Species inquirenda* according to Bauernfeind and Soldán (2012: 199).

27 Several records from the Western and Eastern Mediterranean Region. The record from Corsica is based on OPIE-benthos data. The information published by Belfiore and D’Antonio (1991: 260) [sub. Cloeon gr. simile] needs to be clarified. Recently DNA barcoded by Cardoni et al. (2015).

28 So far known from several localities in Sicily and Sardinia (Belfiore and D’Antonio 1991; Buffagni et al. 2003; Bauernfeind and Soldán 2012). Reported from Elba and Sardinia by Cardoni et al. (2015), with remarks on its possible presence in the Corse-Sardinian biogeographic region; based on DNA barcoding the specimens from Elba and Sardinia may however represent a cryptic endemic species as they differ significantly from specimens of Continental Europe (Cardoni et al. 2015).

29 So far only known from several localities in Rhodos (see Sowa 1985; Sroka et al. 2010); probably endemic to the island (Bauernfeind and Soldán 2012).

30 The record from Sicily by Buffagni et al. (2003) refers to an earlier record by Belfiore et al. (1991: 32) [sub. Pseudocentroptilum sp. gr. pulchrum). According to a tabular summary by Belfiore and D’Antonio (1991). *Procloeon pulchrum* was considered absent from the island. Most probably recorded by Grandi (1966: 327) [sub. *Centroptilum pennulatum* Etn.] from Sicily for the first time. The problem with proper identification of material previously assigned to the *Procloeon pennulatum* species-group is briefly discussed by Belfiore (1988), Belfiore and D’Antonio (1991) and Belfiore et al. (1991). The respective taxonomical status of this material needs to be clarified.

31 The single record from Lesbos [sub. *Pseudocentroptilum motasi* Bogoescu, 1947] by Keffermüller and Sowa (1984: 334−338, figs 53−55) is based on material collected by H. Malicky.

### Annotated checklist of Baetidae in the Mediterranean islands

The history on the mayfly fauna of the Mediterranean islands dates back to the first published observations by Hagen (1864). In this contribution, seven mayfly species were reported from Corsica, including three species of Baetidae. Significant early publications dealing with the Corsican mayfly fauna and also including the description of new species were contributed by Esben Petersen (1912; 1913). All other publications in the early 20^th^ century (Jakobson and Bianki 1905; Lestage 1922; Kimmins 1930) in fact were just compilations and summaries of H.A. Hagen’s and M. Esben-Petersen’s earlier investigations. The first records of the mayfly fauna of the Balearic Islands was published by Navás (1914). Literature on the distribution of Baetidae in the Mediterranean Islands however is scattered.

The annotated checklist presented here (Table 2) provides the first comprehensive compilation of records of Baetidae in the Mediterranean islands incorporating also most recent records and findings along with detailed critical comments on previous records.

## Supplementary Material

XML Treatment for Baetis (Baetis) cypronyx
